# *Listeria monocytogenes* invasion in goat brain tissues: mechanisms of blood–brain barrier disruption and regulation of apoptosis and autophagy

**DOI:** 10.3389/fmicb.2026.1748896

**Published:** 2026-03-02

**Authors:** Yunhai Hu, Lingkang Liu, Wenya Zheng, Ben Liu, Songlin Ding, Siyuan Shi, Yifan Wu, Yu Cao, Jingya Liu, Xiaojie Zhou, Xinli Huang

**Affiliations:** 1 College of Life Science and Resources and Environment, Yichun University, Yichun, Jiangxi, China; 2 Yichun University Research Center for Traditional Chinese Veterinary Medicine and Animal Embryo Engineering Technology, Yichun, Jiangxi, China

**Keywords:** apoptosis, autophagy, blood–brain barrier, brain tropism, goat, *Listeria monocytogenes*

## Abstract

**Background:**

*Listeria monocytogenes* (LM), a zoonotic intracellular pathogen, causes fatal neurological infections in ruminants (e.g., goats) and humans. However, the mechanisms by which LM breaches the blood–brain barrier (BBB) and regulates neuronal programmed cell death (apoptosis/autophagy) remain unclear in caprine models—knowledge that is critical for livestock disease control. This study aimed to investigate the spatiotemporal invasion pathway of LM in goat central nervous system, its brain region-specific effects on apoptosis and autophagy, and the role of the Pink1/Parkin pathway in mitochondrial autophagy during LM infection.

**Methods:**

Goats were intravenously infected with LM to establish an intracranial infection model. Bacterial loads in brain tissues were quantified, and multiple techniques (immunofluorescence, TUNEL, immunohistochemistry, Western blot, qRT-PCR) were used to detect BBB integrity, apoptotic/autophagic markers, and related pathway proteins (E-cadherin/c-Met, Bcl-2/Bax, LC3B, Pink1/Parkin).

**Results:**

LM showed tropism for brainstem regions (midbrain, pons, medulla oblongata) with focal colonization in neurons and glial cells. BBB tight junction proteins (ZO-1, Claudin-1, Occludin) exhibited region-specific dysregulation; notably, an upregulation of Claudin-1 and Occludin was observed in the medulla, suggesting a localized compensatory response. LM infection was associated with the activation of the E-cadherin/c-Met pathway, potentially facilitating transendothelial and neuronal invasion. Apoptosis (Bcl-2/Bax imbalance) and autophagy (LC3B, Pink1/Parkin) were heterogeneously regulated across brain regions, with significant quantitative changes observed in the cerebrum, cerebellum, midbrain, and medulla.

**Conclusion:**

LM invades goat brain tissues coinciding with BBB disruption, exhibits brainstem tropism, and modulates apoptosis and autophagy through region-specific pathways, providing novel insights into LM-induced neurological pathogenesis in ruminants.

## 1 Introduction

*Listeria monocytogenes* (LM) is a facultative intracellular bacterium responsible for listeriosis, a zoonotic disease with severe neurological manifestations in humans and animals (Ravindhiran et al., 2023). Neurolisteriosis is characterized by meningoencephalitis, with mortality rates exceeding 30% in humans and causing significant economic losses in livestock (Hurtado et al., 2017; Wei et al., 2020). Goats, as important ruminant animals, are highly susceptible to neurological listeriosis (rhombencephalitis), a clinical form associated with mortality rates exceeding 50% in infected herds (Dreyer et al., 2016), yet the mechanisms underlying LM neuroinvasion and its impact on brain homeostasis remain underexplored.

The blood–brain barrier (BBB), composed of brain microvascular endothelial cells, tight junctions (TJs), and astrocyte end-feet, is a critical defense against pathogen entry into the central nervous system (CNS) (Profaci et al., 2020). TJs, formed by core proteins including Claudins, Occludin, and ZO-1, are essential for maintaining BBB integrity (Krug and Fromm, 2024; Dithmer et al., 2024; Gu et al., 2021). However, how LM disrupts these structures in goats—an important model for ruminant-specific pathogenesis—remains uncharacterized. In addition, LM’s invasion of host cells is mediated by two key virulence factors: InlA, which binds to the epithelial receptor E-cadherin, and InlB, which interacts with the tyrosine kinase c-Met (Phelps et al., 2018; Osek and Wieczorek, 2022). However, emerging evidence indicates that the invasion mechanism is more complex, involving additional virulence factors such as the *Listeria* adhesion protein (LAP). Recent studies have demonstrated that LAP facilitates barrier disruption and bacterial translocation by modulating tight junction proteins (e.g., occludin and claudin) and the cytoskeletal protein vimentin (Drolia et al., 2018; Ghosh et al., 2018; Drolia and Bhunia, 2019; Liu et al., 2023). While these pathways are well-documented in LM’s intestinal epithelial invasion, their role in facilitating BBB crossing in goats has not been explored. This knowledge gap is critical, as the BBB represents a unique barrier distinct from intestinal epithelia, and understanding its disruption could inform targeted prevention strategies for caprine neuroinfection.

Once inside the CNS, LM manipulates host cell death pathways, including apoptosis and autophagy, to support intracellular survival and replication. Apoptosis, regulated by the Bcl-2 family [with Bcl-2 inhibiting and Bax promoting cell death (Meichner et al., 2016, Takahashi et al., 2008)], and autophagy, a cellular recycling process mediated by LC3B and the Pink1/Parkin mitochondrial autophagy pathway (Lazarou et al., 2015; Nguyen et al., 2016; Harper et al., 2018), are both hijacked by LM in other species. However, no studies have directly tested whether LM drives brain region-specific apoptosis or autophagy in goats—a phenomenon that could explain the localized neurological lesions (e.g., brainstem damage) observed in clinical cases. Specifically, whether LM modulates the Bcl-2/Bax balance or Pink1/Parkin pathway to induce region-specific pathological changes in goat brains remains unaddressed.

To address these critical gaps in veterinary microbiology, we established a goat LM neuroinfection model to investigate: (1) the spatiotemporal pattern of LM invasion and colonization in brain regions; (2) BBB integrity changes and the role of E-cadherin/c-Met in LM transmigration; (3) brain region-specific regulation of apoptosis (Bcl-2/Bax) and autophagy (LC3B, Pink1/Parkin); and (4) the potential involvement of Pink1/Parkin-mediated mitophagy in LM pathogenesis. The findings aim to clarify LM’s neuroinvasive mechanisms and provide a basis for controlling neurological listeriosis in ruminants.

## 2 Materials and methods

### 2.1 Animal model and sample collection

The animal experiment was approved by the Yichun Laboratory Animal Welfare and Ethics Committee (Approval Number: YCU-2025024), following the principles of animal welfare. Twelve healthy adult Ganxi goats (30-month-old, 30–40 kg) were purchased from Yichun Local Black Goat Breeding Co., Ltd., with a 7-day acclimation period before the experiment to exclude pre-existing infections. The goat-derived LM strain used in this study was isolated from a clinical case of caprine neurological listeriosis in our laboratory (serotype 4b, confirmed by multiplex PCR). The infection group (*n* = 6) received a single jugular injection of 10 mL LM suspension (1 × 10^7^ CFU/g); controls (*n* = 6) received 10 mL sterile 0.9% NaCl (Drevets et al., 2004; Oevermann et al., 2010). Animals were monitored daily for clinical signs (lethargy, ataxia, neurological symptoms). When typical neurological signs appeared (72–96 h post-infection), all goats were anesthetized prior to euthanasia via intravenous injection of pentobarbital sodium (60 mg/kg body weight; Sigma-Aldrich, St. Louis, United States) administered through the jugular vein. Euthanasia was then performed by exsanguination of the carotid artery under deep anesthesia, in accordance with the American Veterinary Medical Association (AVMA) Guidelines for the Euthanasia of Animals (2020 edition). Brain tissues (cerebrum, cerebellum, midbrain, pons, medulla oblongata) were collected, with portions fixed in 4% paraformaldehyde (for histology) or stored at −80 °C (for molecular analysis).

### 2.2 Bacterial load quantification

Briefly, the brain tissue homogenates (10% w/v in PBS) were serially diluted and plated on TSA agar. Colonies were counted after 24 h incubation at 37 °C, and results were expressed as CFU/g tissue.

### 2.3 Immunofluorescence staining

Paraffin-embedded sections (5 μm) were deparaffinized in xylene, antigen-retrieved in gradient ethanol, and antigen-retrieved in citrate buffer (pH 6.0, 95 °C for 20 min). Sections were blocked with 5% BSA (Solarbio, Beijing, China) for 1 h at 37 °C, then incubated with primary anti-LM antibody (1:800; Abcam, Cambridge, UK) at 4 °C overnight. After 3 washes with PBS (0.01 M, pH 7.4), sections were incubated with Alexa Fluor® 594-conjugated secondary antibody (1:500; Thermo Scientific, Waltham, United States) for 1 h at 37 °C. Nuclei were stained with DAPI (Beyotime, Shanghai, China) for 5 min. Images were captured using an inverted fluorescence microscope (Ti-S, Nikon, Tokyo, Japan) at 200× magnification, with 5 random fields per section analyzed.

### 2.4 TUNEL assay

Apoptotic cells were detected using the TUNEL BrightGreen Apoptosis Detection Kit (Vazyme) according to the manufacturer’s protocol. TUNEL-positive cells were counted in 5 fields/section and expressed as density (the percentage of TUNEL-positive cells).

### 2.5 Immunohistochemistry

According to the published article (Hong et al., 2024), immunohistochemical staining was performed on the paraffin-embedded brain tissue sections. Sections were incubated with primary antibodies against E-cadherin (1:200; Abcam, Cambridge, UK), c-Met (1:300; Abcam, Cambridge, UK), ZO-1 (1:100; Santa Cruz Biotechnology, Dallas, United States), Claudin-1 (1:200; Santa Cruz Biotechnology, Dallas, United States), Occludin (1:150; Santa Cruz Biotechnology, Dallas, United States), LC3B (1:400; Bioss, Beijing, China), PINK1 (1:200; Bioss, Beijing, China), Parkin (1:200; Bioss, Beijing, China), Bax (1:200; Abcam, Cambridge, UK) and Bcl-2 (1:200; Abcam, Cambridge, UK) at 4 °C overnight. After HRP-conjugated secondary antibody incubation, signals were visualized with 2, diaminobenzidine (DAB, Vector Laboratories, Inc.). The results were observed under an optical microscope.

### 2.6 Western blot analysis

Total proteins from brain tissues were extracted using the total protein extraction kit (Solarbio, Beijing, China) and the protein concentration was determined using the BCA protein assay kit (Boster, Wuhan, China). Equal amounts of protein (50 μg) were separated on a 10% sodium dodecyl sulfate polyacrylamide gel electrophoresis (SDS-PAGE) and transferred to a polyvinylidene fluoride (PVDF) membrane. The membrane was blocked at room temperature for 1 h with 5% skimmed milk powder, then incubated with the primary antibodies at 4 °C overnight, followed by a standard washing procedure and incubation with the secondary antibody at room temperature for 2 h. The dilution ratios of the primary antibodies (Table 1) for different target proteins were as follows: Claudin-1 (1:1000), Occludin (1:1000), Bax (1:1000), Bcl-2 (1:1500), Pink1 (1:1000), Parkin (1:1500), LC3B (1:1500). All horseradish peroxidase (HRP)-conjugated secondary antibodies were purchased from Bioss Biotechnology. The blots were visualized using the ECL ultra-sensitive chemiluminescence detection kit (NCM, Suzhou, China), and the protein bands were displayed using the AI600 multi-functional imaging system (GE Healthcare, United States). The gray value analysis was performed using ImageJ software.

**Table 1:** The information of the primary antibodies.

Antibodies	Source	Identifier
Mouse monoclonal anti-E-cadherin	Abcam	Cat# ab76055, RRID: AB_1310159
Rabbit monoclonal anti-c-Met	Abcam	Cat# ab225524
Rat monoclonal anti-ZO-1	Santa Cruz Biotechnology	Cat# sc-33725, RRID: AB_628459
Mouse monoclonal anti-Claudin-1	Santa Cruz Biotechnology	Cat# sc-166338, RRID: AB_2244863
Mouse monoclonal anti-Occludin	Santa Cruz Biotechnology	Cat# sc-133256, RRID: AB_2156317
Rabbit monoclonal anti-LC3B	Bioss	Cat#bsm-60842R
Rabbit monoclonal anti-PINK1	Bioss	Cat#bs-22173R
Rabbit polyclonal anti-Parkin	Bioss	Cat# bs-23687R
Rabbit monoclonal anti-Bax	Abcam	Cat# ab32503, RRID: AB_725631
Rabbit monoclonal anti-Bcl-2	Abcam	Cat# ab182858, RRID: AB_2715467

### 2.7 Quantitative reverse transcription PCR (qRT-PCR)

Total RNA was extracted using TRIzol reagent (Invitrogen, Carlsbad, CA, United States) and reverse-transcribed into cDNA using the miScript reverse transcription kit (Vazyme, Nanjing, China). qRT-PCR was performed using ChamQ SYBR qPCR premix (Vazyme, Nanjing, China) on a StepOnePlus system (Applied Biosystems) (Table 2). Primer sequences for target genes (E-cadherin, c-Met, Bcl-2, Bax, LC3B, Pink1, Parkin) and reference gene (β-actin) are listed in Supplementary Table S1. Relative expression was calculated using the 2^−^ΔΔCt method.

**Table 2:** Primer sequences for quantitative real-time PCR (qRT-PCR).

Gene	Primer sequence(5′ → 3′)	GeneBank accession	Product length (bp)
*ZO-1*	F: CTTCCCGGACTTTTGTCCCA R: CCACCGTCCGCATAAACATC	NM_001163574.2	108
*Claudin-1*	F: GCCATCTACGAGGGACTGTG R: CCCCAGCAGGATGCCAATTA	NM_016674.4	141
*Occludin*	F: CTCTTTCCTTAGGCGACAGC R: ACATGGCTGATGTCACTGGT	NM_001360536.1	148
*E-cadherin*	F: TCGGAGGAGAAGGGTGGTCAAAG R: CTGGCTCAAGTCAAAGTCCTGGTC	NM_001317185.2	121
*c-Met*	F: TGTGTGCGATTGGAGGAATGCC R: AAAGTCCCAGCCACAAACAGTCAG	NM_001317186.1	132
*Pink1*	F: TCATCCAGCGAAGCCATCTTTAGC R: TCCCTTGGGTCTTCCGTGAGTG	XM_018055041.1	108
*Parkin*	F: GGAGGTGGTTGCTAAGCGACAG R: ATGTGAACGATGCTCTGCTGATCC	XM_018053444.1	86
*LC3B*	F: AGAAGGCGCTTACAGCTCAATGC R: ACTTCACAAATCGGAGTGGACACAC	XM_018061829.1	92
*Bax*	F: GGCCCTTTTGCTTCAGGGTT R: CAGACACTCGCTCAGCTTCT	XM_018062750.1	121
*Bcl-2*	F: GAGTTCGGAGGGGTCATGTG R: TACAGCTCCACAAAGGCGTC	NM_001314213.1	152
*β-actin*	F: GGAGGTGGTTGCTAAGCGACAG R: ATGTGAACGATGCTCTGCTGATCC	NM_001314342.1	177

### 2.8 Statistical analysis

Data were analyzed using GraphPad Prism 10.1. Differences between groups were assessed by unpaired *t*-test. Results are presented as mean ± SD. *p* < 0.05 (*) was considered statistically significant, *p* < 0.01 (**) as highly significant, and *p* > 0.05 (ns) indicates no significant difference.

## 3 Results

### 3.1 LM colonization and tropism in goat brain tissues

**Figure 1:**
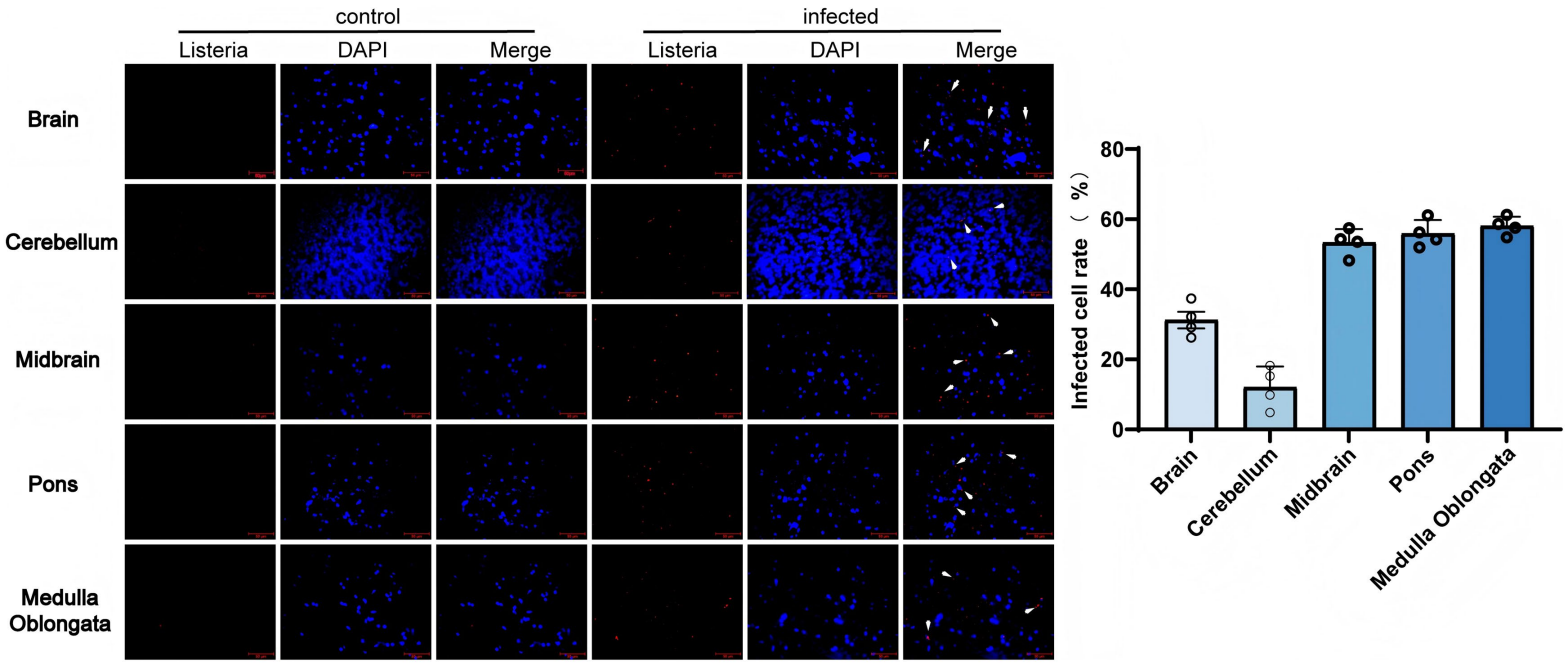
Distribution and tropism of *Listeria monocytogenes* (LM) in goat brain tissues detected by immunofluorescence. Red fluorescent signals (white arrow) indicate intracellular LM; blue signals indicate cell nuclei. Scale bar: 20 μm.

Clinical signs in infected goats included ataxia, head tilt, and lethargy within 72–96 h post-infection. Successful establishment of systemic infection was confirmed by characteristic pathological lesions in visceral organs, as detailed in our previous report using this model (Wu et al., 2019). Specifically, infected animals exhibited multifocal hepatic necrosis and splenic inflammatory cell infiltration, verifying the hematogenous dissemination of the pathogen. To objectively assess the distribution of *Listeria monocytogenes* (LM), we performed a quantitative analysis of bacterial signals across multiple brain regions (Figure 1). Immunofluorescence showed LM was localized in the cytoplasm of neurons (NeuN^+^) and glial cells (GFAP^+^), with focal aggregation. Quantitative analysis demonstrated significantly higher bacterial densities in the midbrain, pons, and medulla oblongata (*p* < 0.01) compared to the brain and cerebellum, indicating a marked infection tropism for the brainstem. Quantitative culture revealed an average LM load of (6.52 ± 0.83) × 10^7^ CFU/g in infected brain tissue, with colony density showing a significant positive correlation with LM fluorescence intensity (r = 0.89, *p* < 0.01). These data indicate that in this caprine infection model, LM is associated with blood–brain barrier disruption and subsequent CNS colonization, exhibiting a characteristic predilection for the brainstem.

### 3.2 BBB integrity disruption

**Figure 2:**
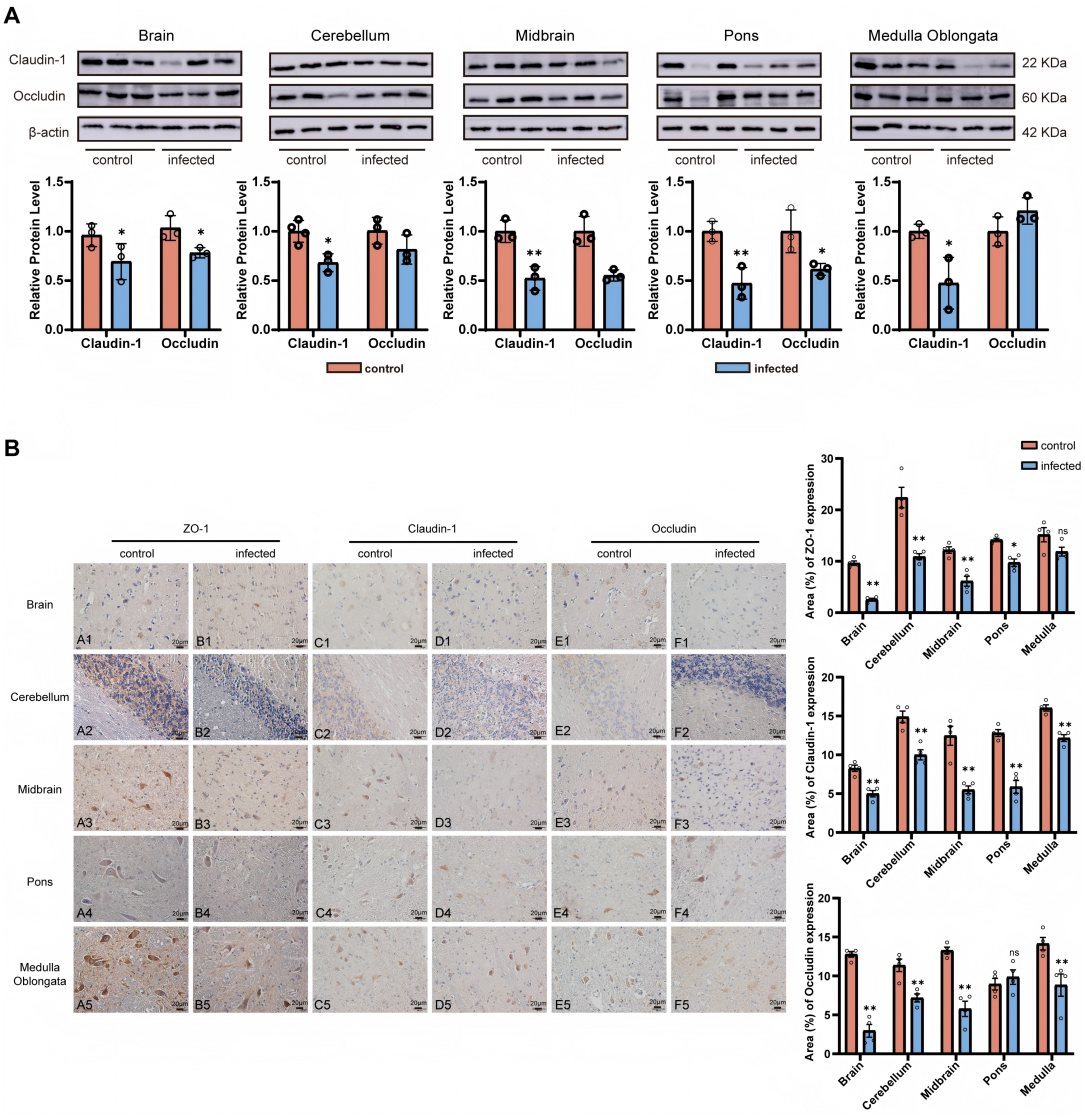
*Lm* infection leads to the disruption of the blood–brain barrier integrity in goats. **(A)** Western blot analysis of Claudin-1 and Occludin protein expression in different brain regions. Top: Representative blots (Claudin-1: 22 kDa; Occludin: 60 kDa; β-actin: 42 kDa, internal reference). Bottom: Quantitative analysis of relative protein expression (mean ± SE, *n* = 6). **(B)** Immunohistochemical (IHC) localization of ZO-1, Claudin-1, and Occludin in goat brain tissues. Scale bar: 20 μm. **(C)** qRT-PCR analysis of *ZO-1*, *Claudin-1*, and *Occludin* mRNA expression (mean ± SE, *n* = 6). Statistical notation: *P* < 0.01 (**), *P* < 0.05 (*), *P >* 0.05 (ns); unpaired *t*-test.

To further evaluate the impact of LM-induced neuroinvasion on blood–brain barrier (BBB) integrity, we examined the expression of tight junction (TJ) proteins (ZO-1, Claudin-1, and Occludin) using immunohistochemistry and Western blotting. Western blot analysis revealed regional heterogeneity in TJ protein levels (Figure 2A): Claudin-1 and Occludin expression in the cerebrum, midbrain, and pons of the infected group was significantly lower than that of the control group (*p* < 0.05), while a significant increase was observed in the medulla oblongata (*p* < 0.05). Quantitative immunohistochemistry (IHC) analysis further substantiated these regional differences (Figure 2B). Claudin-1 expression was significantly reduced across all examined brain regions (*p* < 0.01). For ZO-1 and Occludin, significantly reduced signal intensity and structural continuity were observed in most regions (*p* < 0.05), with the exception of ZO-1 in the medulla and Occludin in the pons, where no statistically significant changes were detected (*p* < 0.05). In the control group, TJ proteins were widely distributed in neurons and glial cells, with ZO-1 primarily localized to cortical astrocytes and cerebellar Purkinje cells. Following infection, the structural continuity of these proteins was markedly disrupted in the cerebral cortex, cerebellum, midbrain, and pons.

These findings suggest that LM infection is associated with region-specific dysregulation of BBB tight junction proteins, potentially compromising the integrity of the blood–brain barrier.

### 3.3 Activation of E-cadherin/c-Met pathway

**Figure 3:**
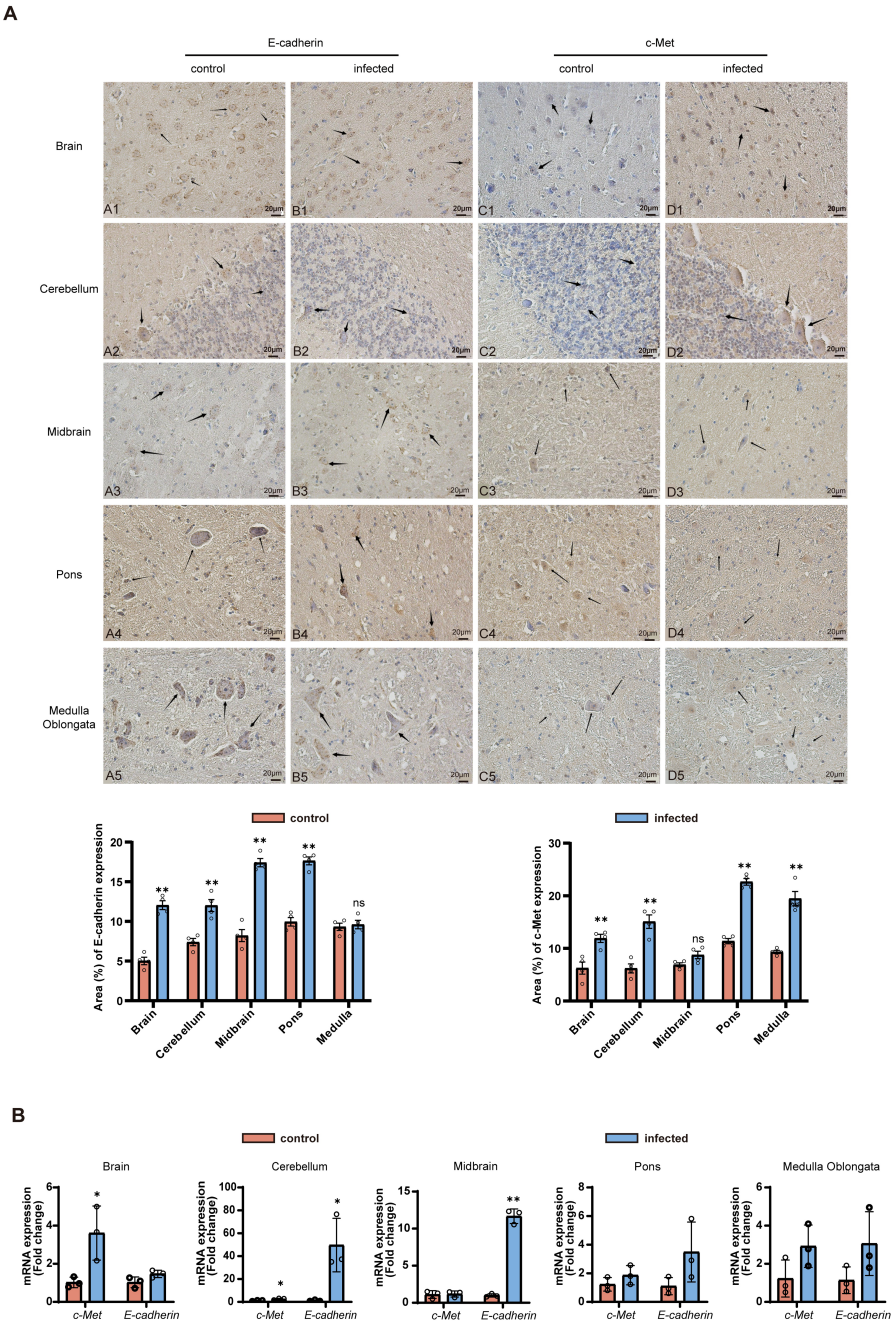
LM activates the E-cadherin/c-Met pathway to promote transendothelial and neuronal invasion in goat brains. **(A)** IHC localization of E-cadherin and c-Met in goat brain tissues. Scale bar: 20 μm. **(B)** qRT-PCR analysis of *E-cadherin* and *c-Met* mRNA expression (mean ± SE, *n* = 6). Statistical notation: *P* < 0.01 (**), *P* < 0.05 (*), *P* > 0.05 (ns); unpaired *t*-test. Arrows indicate cells exhibiting positive expression of E-cadherin and c-Met.

To objectively evaluate the involvement of the E-cadherin/c-Met pathway during LM infection, we performed a comprehensive quantitative analysis of their protein and mRNA levels (Figure 3). Immunohistochemical analysis revealed that both E-cadherin and c-Met were widely distributed in the cell membranes and cytoplasm of neurons and glial cells in both infected and control goat brain tissues (Figure 3A). Notably, E-cadherin exhibited characteristic high expression in cortical astrocytes, cerebellar Purkinje cells, and brainstem trigeminal ganglia, while c-Met was enriched in brainstem vascular endothelial cells. Quantitative IHC scoring demonstrated that E-cadherin expression was significantly upregulated in the brain, cerebellum, midbrain, and pons (*p* < 0.01), with the exception of the medulla oblongata, where no significant change was observed. Similarly, c-Met expression was significantly increased in the brain, cerebellum, pons, and medulla oblongata (*p* < 0.01), while no statistically significant change was detected in the midbrain.

qRT-PCR analysis further substantiated these findings at the transcriptional level (Figure 3B): *c-Met* mRNA in the brain, cerebellum, midbrain, and brainstem of the infected group was significantly upregulated (*p* < 0.05), with an increase also observed in the medulla oblongata; *E-cadherin* mRNA was significantly upregulated in the brain and midbrain (*p* < 0.05), while there was a non-significant increase in other regions (*p* > 0.05).

These findings indicate that LM infection is associated with the synergistic enhancement of the E-cadherin/c-Met pathway at both the transcriptional and protein levels. This synergy may be linked to the pathogen’s ability to interact with host endothelial and neuronal cells during central nervous system (CNS) colonization.

### 3.4 Brain region-specific apoptosis

**Figure 4:**
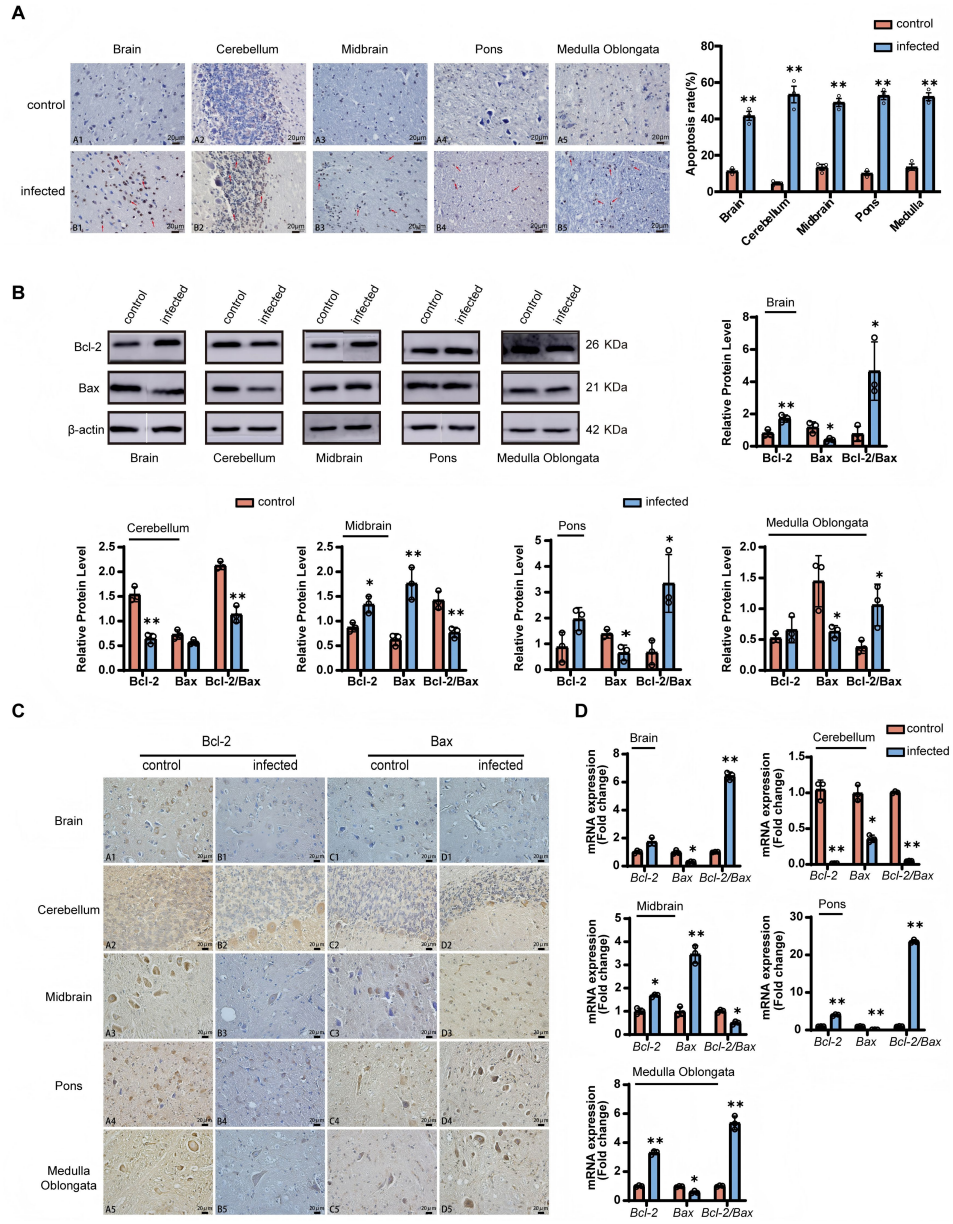
LM induces region-specific apoptosis in goat brains via regulating the Bcl-2/Bax balance. **(A)** TUNEL assay for apoptotic cells. **(B)** Western blot analysis of Bcl-2, Bax, and Bcl-2/Bax ratio (mean ± SE, *n* = 6). **(C)** IHC localization of Bcl-2 and Bax. Scale bar: 20 μm. **(D)** qRT-PCR analysis of Bcl-2, Bax, and Bcl-2/Bax mRNA (mean ± SE, *n* = 6). Statistical notation: *P* < 0.01 (**), *P* < 0.05 (*), *P* < 0.05 (ns); unpaired *t*-test. Arrows indicate TUNEL-positive cells (brown nuclei).

To objectively assess the extent of programmed cell death, a quantitative analysis of TUNEL-positive cells was performed across multiple brain regions (Figure 4A). Statistical analysis revealed that the density of apoptotic cells (measured as the percentage of TUNEL-positive cells) was significantly higher in the infected group compared to the control group across the brain, cerebellum, midbrain, pons, and medulla oblongata (*p* < 0.01). Representative high-magnification images clearly illustrated typical apoptotic features, including nuclear shrinkage, chromatin marginalization, and the formation of apoptotic bodies (indicated by arrows in Figure 4A).

Western blot quantification (Figure 4B) confirmed: the Bcl-2 protein was significantly upregulated in the brain and midbrain of the infected group (*p* < 0.05), and significantly downregulated in the cerebellum (*p* < 0.01); the Bax protein was specifically upregulated in the midbrain (*p* < 0.01), and significantly downregulated in other brain regions (*p* < 0.05). The Bcl-2/Bax ratio was significantly increased in the brain, midbrain and medulla oblongata (*p* < 0.01), and significantly decreased in the cerebellum and midbrain (*p* < 0.05).

Immunohistochemical analysis indicated (Figure 4C) that Bcl-2 and Bax were widely co-localized in the cytoplasm of neurons, and showed strong positive signals in cortical pyramidal cells and cerebellar Purkinje cells. After infection, the expression intensity of Bcl-2 in motor neurons of the medulla oblongata was significantly upregulated (*p* < 0.01), while Bax was significantly downregulated in the cerebellar granular layer (*p* < 0.01).

qRT-PCR verification (Figure 4D) showed: *Bcl-2* mRNA was significantly upregulated in the midbrain and pons (*p* < 0.01), and significantly downregulated in the cerebellum (*p* < 0.01); *Bax* mRNA was specifically upregulated in the midbrain (*p* < 0.01), and significantly decreased in other brain regions (*p* < 0.05); the transcription ratio of *Bcl-2*/*Bax* was highly consistent with the changes in protein levels.

These results indicate that LM infection can regulate the Bcl-2/Bax balance in a region-specific manner, and induce the activation of the apoptotic pathway in brain tissues.

### 3.5 Dysregulation of autophagy pathways

**Figure 5:**
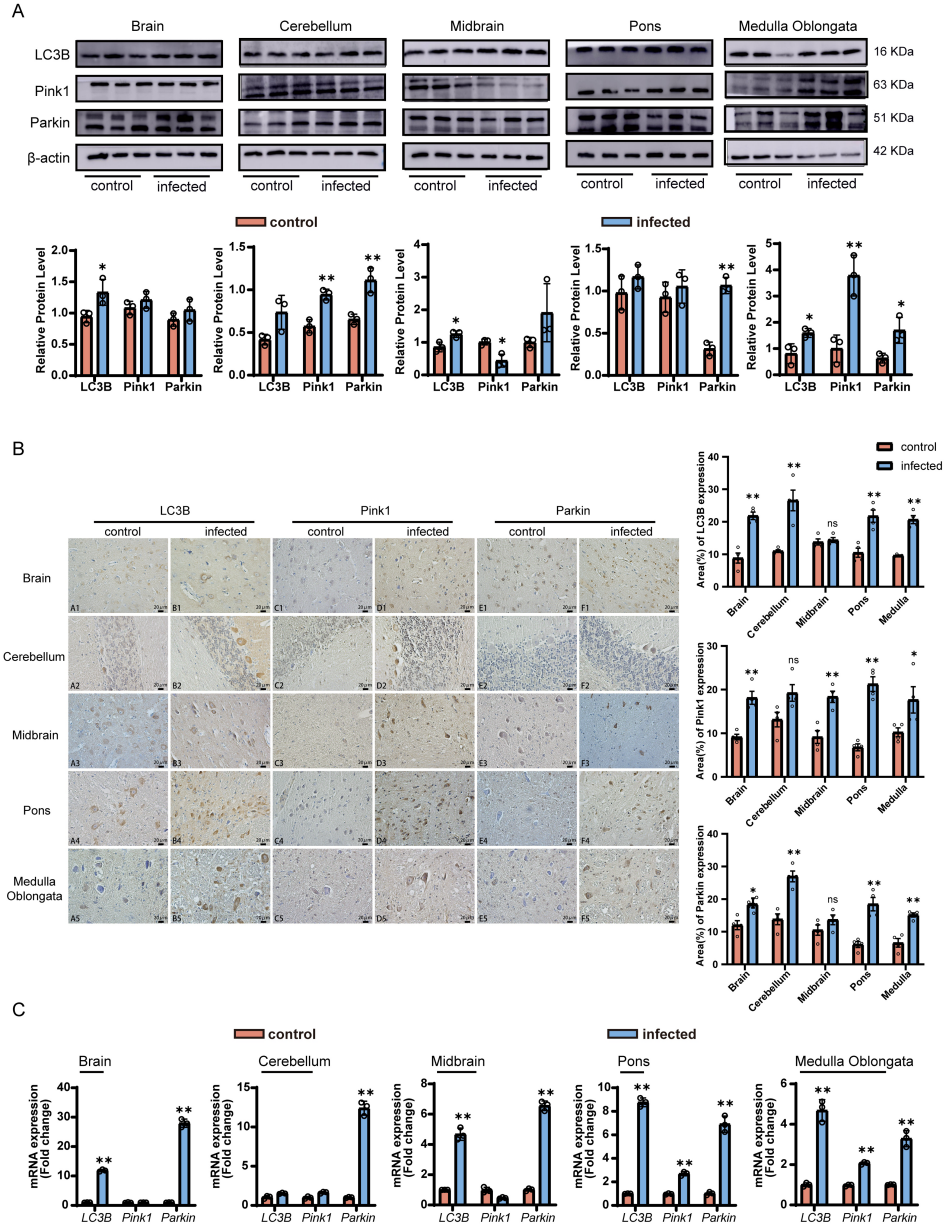
*Listeria monocytogenes* (LM) infection induces abnormal activation of autophagy pathways in goat brains. **(A)** Western blot analysis of LC3B, Pink1, and Parkin protein expression (mean ± SE, *n* = 6). Top: Representative blots (LC3B: 16 kDa; Pink1: 63 kDa; Parkin: 51 kDa; β-actin: 42 kDa, internal reference). Bottom: Quantitative analysis. **(B)** IHC localization of LC3B, Pink1, and Parkin. Scale bar: 20 μm. **(C)** qRT-PCR analysis of *LC3B*, *Pink1*, and *Parkin* mRNA (mean ± SE, *n* = 6). Statistical notation: *P* < 0.01 (**), *P* < 0.05 (*), *P* > 0.05 (ns); unpaired *t*-test.

To assess the potential involvement of autophagy during LM infection, we analyzed the expression of total LC3B, Pink1, and Parkin (Figure 5). Western blot quantification (Figure 5A) revealed significantly elevated LC3B protein levels in the brain, midbrain, and medulla oblongata of the infected group (*p* < 0.05), indicating enhanced autophagosome formation. Pink1 protein was significantly up-regulated in the cerebellum and medulla (*p* < 0.01) but down-regulated in the midbrain (*p* < 0.05). Parkin protein was significantly up-regulated in the cerebellum, pons, and medulla (*p* < 0.01).

Immunofluorescence revealed the co-localization of LC3B, Pink1, and Parkin within the neuronal cytoplasm (Figure 5B). Specifically, LC3B was predominantly localized in cerebellar Purkinje cells, granule cells, and Golgi cells, while Pink1 and Parkin were similarly enriched in Purkinje cells. Quantitative immunohistochemical analysis demonstrated that, with the exception of non-significant changes in LC3B in the midbrain, Pink1 in the cerebellum, and Parkin in the midbrain (*p* >0.05), the expression of LC3B, Pink1, and Parkin was significantly up-regulated in most brain regions (*p* < 0.05). Notably, in the infected group, the positive signal density of these proteins significantly increased in glial cells of the pons and medulla (*p* < 0.01), indicating a robust autophagic stress response in both neuronal and non-neuronal cells.

qRT-PCR verification (Figure 5C) indicated that *LC3B* mRNA was significantly upregulated in the brain, midbrain, and medulla oblongata (*p* < 0.05), and was highly positively correlated with the protein level (*r* = 0.927); *Pink1* mRNA was extremely significantly upregulated in the medulla oblongata and pons (*p* < 0.01), and showed a downward trend in the midbrain; *Parkin* mRNA was extremely significantly upregulated in all brain regions (*p* < 0.01), suggesting a global activation at the transcriptional level.

## 4 Discussion

This study demonstrates that intravenous LM infection in goats results in CNS invasion with distinct brainstem tropism, coincides with compromised BBB integrity via region-specific regulation of tight junctions, and modulates apoptosis and autophagy through brain region-dependent pathways. These findings advance our understanding of LM neuro pathogenesis in ruminants.

First, our identification of LM’s brainstem tropism (midbrain/pons/medulla oblongata) aligns with clinical observations of rhombencephalitis in goats (Dreyer et al., 2016). The observed tropism of LM for the brainstem (midbrain, pons, medulla) aligns with previous reports of LM’s predilection for CNS regions involved in vital functions (Jaguezeski et al., 2018). The high bacterial load in these areas likely contributes to neurological signs (e.g., ataxia) by impairing motor and sensory signaling, highlighting a potential link between LM tropism and clinical manifestations. This tropism likely explains the severe neurological signs (ataxia, head tilt) observed in infected goats, as the brainstem controls essential motor and sensory signaling. Importantly, this finding refines our understanding of LM’s tissue-specific virulence in ruminants: unlike in mice—where LM causes widespread CNS lesions (Seele et al., 2021)—goats exhibit targeted brainstem damage, highlighting the need for species-specific research to avoid extrapolating findings from non-ruminant models.

Second, BBB disruption, a hallmark of LM neuroinvasion, was characterized by dysregulated tight junction proteins (Meireles et al., 2024). Notably, LM’s association with of the BBB via region-specific TJ protein dysregulation reveals a previously unrecognized host response pattern: while Claudin-1 and Occludin were downregulated in the cerebrum, midbrain, and pons (consistent with BBB breakdown), these proteins were upregulated in the medulla (*p* < 0.05), suggesting a localized compensatory response. Our findings regarding TJ disruption align with emerging evidence involving other virulence factors beyond InlA and InlB. Recent studies have highlighted that the Listeria adhesion protein (LAP) interacts with host Hsp60 to activate MLCK, leading to the opening of tight junctions and paracellular translocation (Drolia et al., 2018; Liu et al., 2023). Additionally, surface proteins such as vimentin have been identified as critical receptors facilitating LM invasion into the brain (Ghosh et al., 2018). Therefore, the downregulation of Occludin and Claudin-1 observed in this study likely reflects a cumulative effect of these multifactorial mechanisms, where LAP and other factors may synergistically compromise barrier integrity to facilitate invasion. This “damage-repair” duality echoes observations in sepsis models, where TJ protein changes modulate immune cell infiltration to mitigate brain injury (Gu et al., 2021), but is the first report of such a response in LM-infected ruminants. This “damage-repair” duality warrants further investigation to identify regulatory factors specific to the medulla.

Third, in the early stage of LM infection, it mainly relies on the non-specific immune clearance mediated by the host’s inflammation. Once it invades the cells, it depends on the specific cellular immunity mediated by the host’s lymphocytes (Carrero et al., 2006; Corr and O’Neill, 2009). Through TUNEL cell apoptosis detection, we observed that the number and degree of apoptotic cells in the goat’s brain, cerebellum and midbrain significantly increased after infection with *Listeria monocytogenes*. This indicates that LM infection induced the apoptosis of some brain cells, potentially affecting the brain’s homeostasis and its normal functions. The “execution protease” Caspase-3 can degrade various substrates in the cytoplasm and nucleus, thereby causing cell death (Xie et al., 2016). As the upstream effector factors of Caspase-3, Bax and Bcl-2, their mitochondrial pathway mediated by it is a hallmark of intrinsic apoptosis. Apoptotic stress drives the oligomerization of Bax/Bak, leading to the rupture of the mitochondrial membrane (Zhuang et al., 2021; Bock and Tait, 2020), activating the downstream caspase cascade reaction, and thereby initiating cell apoptosis (Dewson et al., 2009; Subburaj et al., 2015). While the anti-apoptotic protein Bcl-2 maintains cell survival by inhibiting the pro-apoptotic effect of Bax. Our findings that LM-induced apoptosis showed striking regional heterogeneity. The Bcl-2/Bax ratio exhibited dual phenotypes: anti-apoptotic in the cerebrum and medulla (supporting LM’s intracellular persistence) and pro-apoptotic in the cerebellum and midbrain (facilitating bacterial release). Similarly, autophagy markers showed heterogeneity: LC3B (a key autophagosome marker) was upregulated in the cerebrum, midbrain, and medulla (*p* < 0.05), while Pink1/Parkin (mitophagy regulators) were upregulated in the cerebellum/medulla but downregulated in the midbrain (*p* < 0.05). This indicates that LM may modulate host cell apoptosis to ensure its own survival, although this ability is limited by tissue tropism and other factors. For example, intracellular parasitic bacteria such as *Shigella*, *Mycobacterium tuberculosis*, and *Leishmania* inhibit host cell apoptosis by mediating the overexpression of anti-apoptotic proteins Bcl-2 and Bcl-xL or by secreting related virulence factors (Carneiro et al., 2009; Stutz et al., 2021; Ornelas-Cruces et al., 2025).

Fourth, the upregulation of E-cadherin/c-Met pathways in infected brains supports their conserved role in LM invasion, as observed in placental and endothelial cells (Disson et al., 2008). This finding indicates that InlA/E-cadherin and InlB/c-Met pathways are activated during infection in goats, though their relative contribution to BBB crossing requires further verification using receptor-blocking or gene-knockout assays.

This study has several limitations that should be addressed in future work. While this study characterizes the neuroinvasive features of LM in goats and correlates them with the Pink1/Parkin pathway, specific mechanistic causality remains to be firmly established. Subsequent studies should employ *in vivo* gene knockout (e.g., CRISPR-Cas9 knockout of E-cadherin or Pink1) or receptor blocking to strengthen the evidence of key indicators. Previous reports have suggested that apoptosis and autophagy may interact, and the combination of autophagy and apoptosis may be triggered by common upstream signals, which means that the apoptotic and autophagic mechanisms share a common pathway for connecting or polarizing cellular responses (Maiuri et al., 2007; Lindqvist and Vaux, 2014). Although we identified *L. monocytogenes* within GFAP-positive astrocytes and observed inflammatory infiltration, we did not quantitatively assess the specific activation states of microglia and astrocytes (e.g., via Iba1 staining) due to the specific scope of the current study. In addition, a comprehensive study on the connections between neuroinflammation, BBB dysfunction, oxidative stress, autophagy, and apoptosis during *Listeria monocytogenes* infection still needs to be conducted. Further evaluation of the relationship network of these pathways through research and clinical trials may inspire the key pathological pathways of LM and potential prevention strategies.

In conclusion, this study advances our understanding of LM’s neuropathogenesis in goats by uncovering region-specific patterns of invasion, BBB disruption, and cell death regulation. These findings not only fill critical knowledge gaps in ruminant microbiology but also provide actionable targets for controlling neurological listeriosis—ultimately improving livestock health and reducing economic losses in the ruminant industry.

## Data Availability

The datasets presented in this study can be found in online repositories. The names of the repository/repositories and accession number(s) can be found in the article/Supplementary material.
