# Supplementary material for: Listeria monocytogenes invasion in goat brain tissues: mechanisms of blood–brain barrier disruption and regulation of apoptosis and autophagy
Source: Front Microbiol. 2026 Mar 2;17:1748896. doi: 10.3389/fmicb.2026.1748896 (PMC12990321; doi:10.3389/fmicb.2026.1748896)

**Western Blot Instructions and Original Images**

At first, we check the full films of western blot for the protein samples of rooster testicles to ensure the specificity of antibodies. The results were showed as follows:


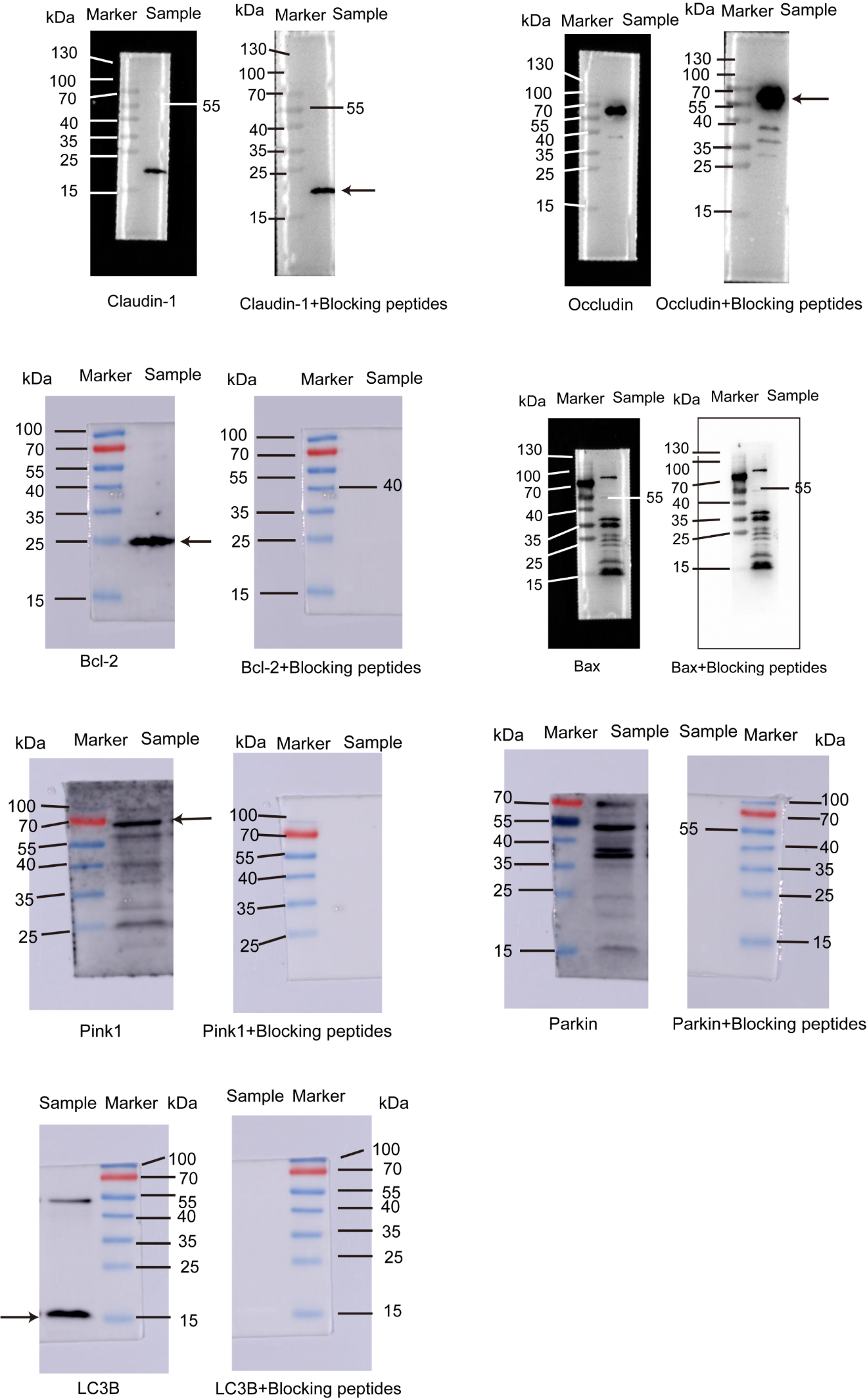


After ensuring the specificity of the anti-Claudin-1, anti-Occludin ,anti-Bax, anti-Bcl-2, anti-Parkin, anti-Pink1 and anti-LC3B, we performed following experiments in article with cropped gels. All original images were showed as follows:

Western blot original images of the Claudin-1 and Occludin proteins in the testes of figure 2.


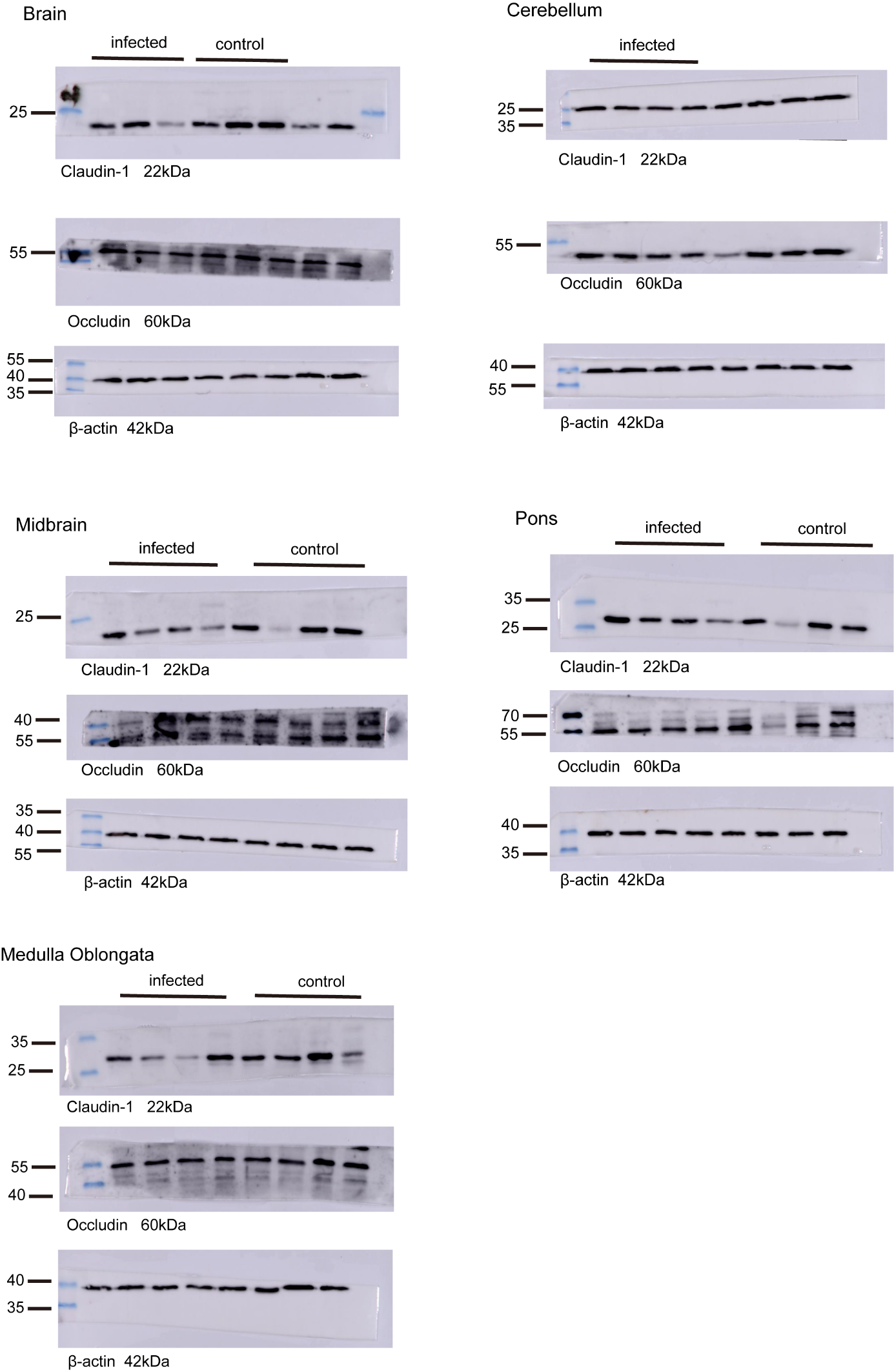


Western blot original images of the Bax and Bcl-2 proteins in the testes of figure 4.


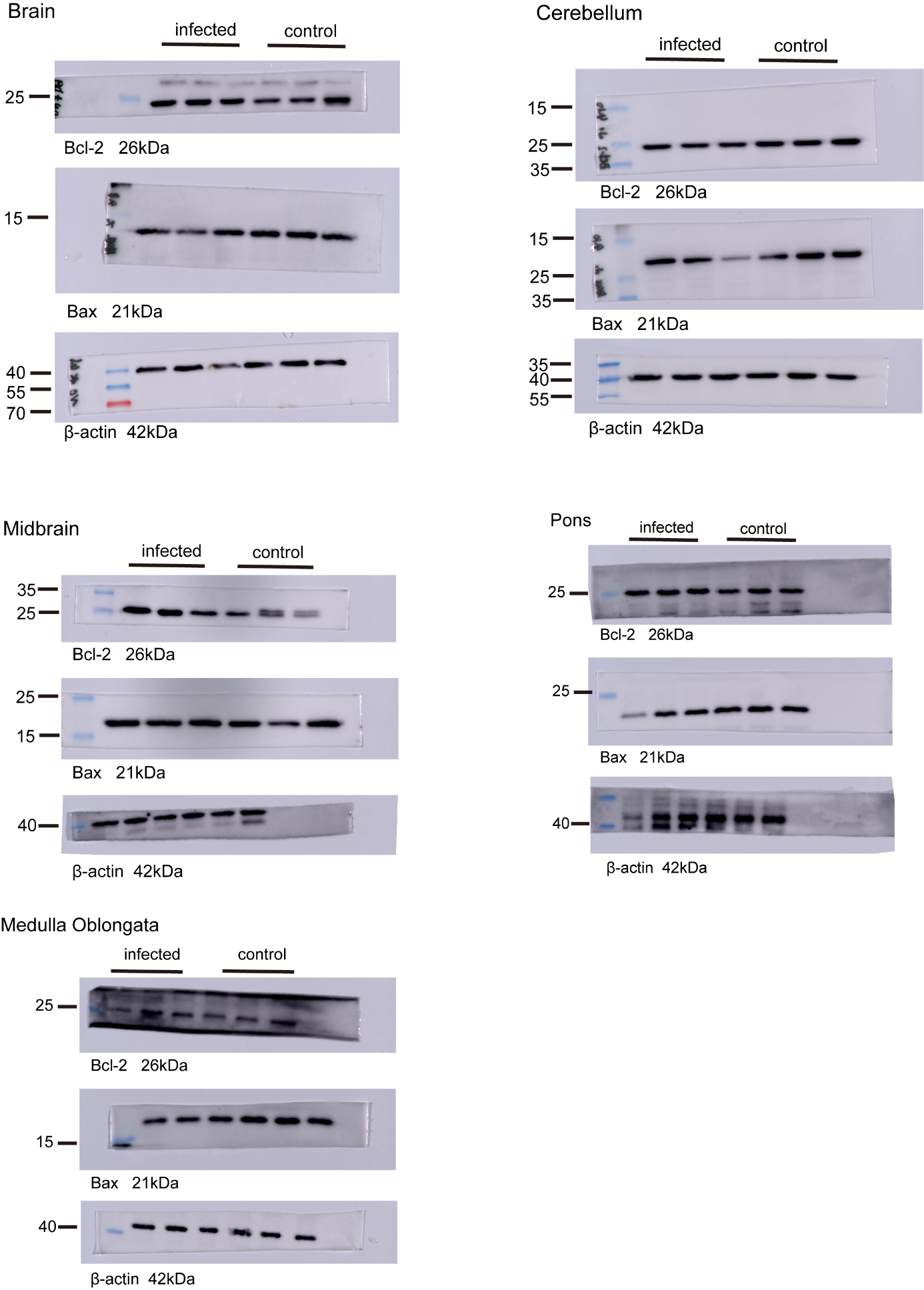


Western blot original images of the Parkin, Pink-1 and Bcl-2 proteins in the testes of figure 5.


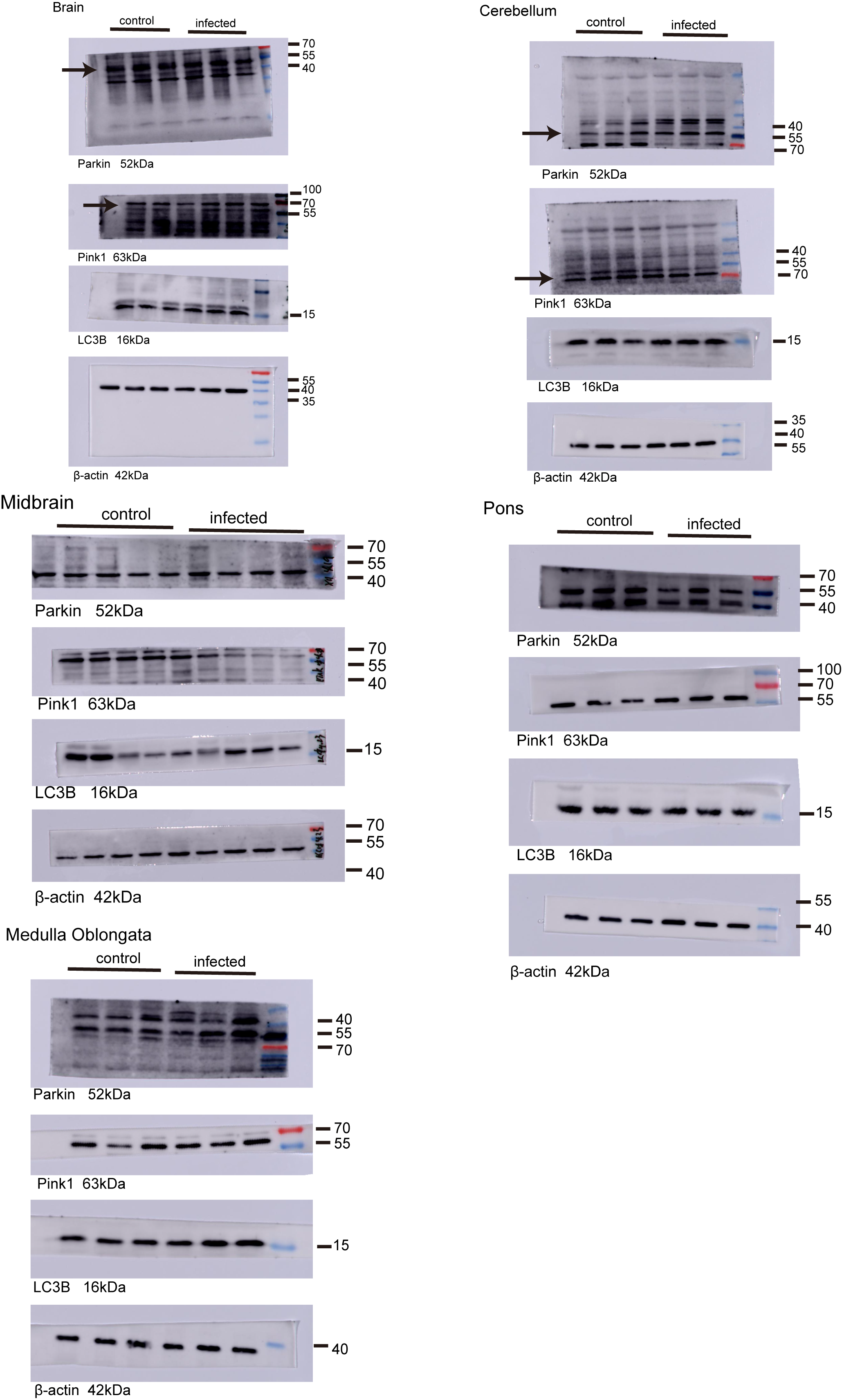

Supplement: Supplementary file 1 [file Table_1.doc]
